# 

**Published:** 1887-02

**Authors:** 


### CONTENTS:

ARTICLE I—Protoplasmic Nutrition and Molecular Metamorphosis in the Dental Tissues. By Alton H. Thompson, D. D. S ..... 433
" II—Professional Courtesies in Connection with what are Commonly Considered Failures in Dental Practice. By C. E. Francis, D. D. S.......... 443
" III—Requirements of Teeth Extraction During the Period of Youth. By J. Hardman, D. D. S .......... 448
" IV—Surgical Diseases of the Tongue. By H. A. Smith, D. D. S. 454
" V—Man's Lost Incisors. By Bertram C. A. Windle, M. A., M. D., and John Humphreys, L. D. S I.......... 458

### EDITORIAL, ETC.

An International Dental Congress.......... 472
Annual Commencement of the University of Maryland, Dental Department.......... 473

### BIBLIOGRAPHICAL.

Transactions of the American Dental Association.......... 474
Caulk's Dental Annual for 1886-87.......... 474
The Dental Office and Laboratory.......... 474
The Dental Review.......... 474
James Vick's Floral Guide for 1887.......... 474
The Western Dental Journal.......... 475

### OBITUARY.

Dr. Frank P. Abbot.......... 475

### MONTHLY SUMMARY.

Restoring a Bicuspid.......... 478
Remedy for Tired Eyes.......... 479
Local Remedy for Neuralgia.......... 480
A Disinfectant.......... 480

